# Data on effect of electrospinning conditions on morphology and effect of heat-treatment temperature on the cycle and rate properties of core-shell LiFePO_4_/FeS/C composite fibers for use as cathodes in Li-ion batteries

**DOI:** 10.1016/j.dib.2019.104364

**Published:** 2019-08-13

**Authors:** Rattiya Hongtong, Panya Thanwisai, Rattakarn Yensano, Jeffrey Nash, Sutham Srilomsak, Nonglak Meethong

**Affiliations:** a Materials Science and Nanotechnology Program, Department of Physics, Faculty of Science, Khon Kaen University, Khon Kaen 40002, Thailand; b Institute of Nanomaterials Research and Innovation for Energy (IN-RIE), Research Network of NANOTEC- KKU (RNN), Khon Kaen University, Khon Kaen 40002, Thailand; c The Graduate School, Udon Thani Rajabhat University, Udon Thani 41000, Thailand

**Keywords:** Lithium-ion batteries, Lithium iron phosphate, Electrospinning, Nanofiber, One-dimensional nanostructures, Morphology

## Abstract

The data in this study are related to the research article “Core-shell electrospun and doped LiFePO_4_/FeS/C composite fibers for Li-ion batteries” [1]. Core-shell LiFePO_4_/FeS/C composites fiber were prepared via an electrospinning method for use as cathodes in Li-ion batteries. The data presented in this paper showed the effect of electrospinning parameters, including applied voltage, solution flow rate, the concentration of polyvinylpyrrolidone (PVP) (wt%) and a mixed PVP/PEO (polyethylene oxide) (w/w%) polymers on the morphological properties of composites fibers. These data were developed using scanning electron microscopy (SEM). Then, the effect of heat-treatment temperature on fiber morphology was investigated using transmission electron microscopy (TEM). The voltage profile and cycle rate properties of the core-shell LiFePO_4_/FeS/C composites obtained after various heat treatments were studied.

**Table:** Specifications Table

Subject area	Materials Science
More specific subject area	Nanomaterials for lithium-ion batteries
Type of data	Figures and table
How data was acquired	SEM, TEM, cycle and rate properties (Swagelok type cells)
Data format	Analyzed data
Experimental factors	Applied voltage, solution flow rate, concentration of PVP, ratios of PVP/PEO, and heat-treatment temperature
Experimental features	Morphology, diameter distribution, cycle and rate properties influenced by heat-treatment temperature
Data source location	Materials Science and Nanotechnology Program, Department of Physics, Faculty of Science, Khon Kaen University, Khon Kaen, Thailand
Data accessibility	Data is within this article
Related research article	[1] R. Hongtong, P. Thanwisai, R. Yensano, J. Nash, S. Srilomsak, N. Meethong, Core-shell electrospun and doped LiFePO_4_/FeS/C composite fibers for Li-ion batteries. https://doi.org/10.1016/j.jallcom.2019.07.008

**Table:** 

**Value of the data** • This research provides a better understanding of the effects of electrospinning parameters on the morphological features and fiber diameters of electrospun LiFePO_4_/FeS/C composites. • Data from this work reveal the appropriate heat-treatment temperatures to obtain unique core-shell electrospun LiFePO_4_/FeS/C composites. • Data from this work show the effect of heat-treatment temperature upon the voltage profile and cycle rate properties of core-shell electrospun LiFePO_4_/FeS/C composites. • These data provide strategies to control the morphological features of other composites fibers that may be considered for use as electrode materials.

## 1 Data

**Fig. 1:**
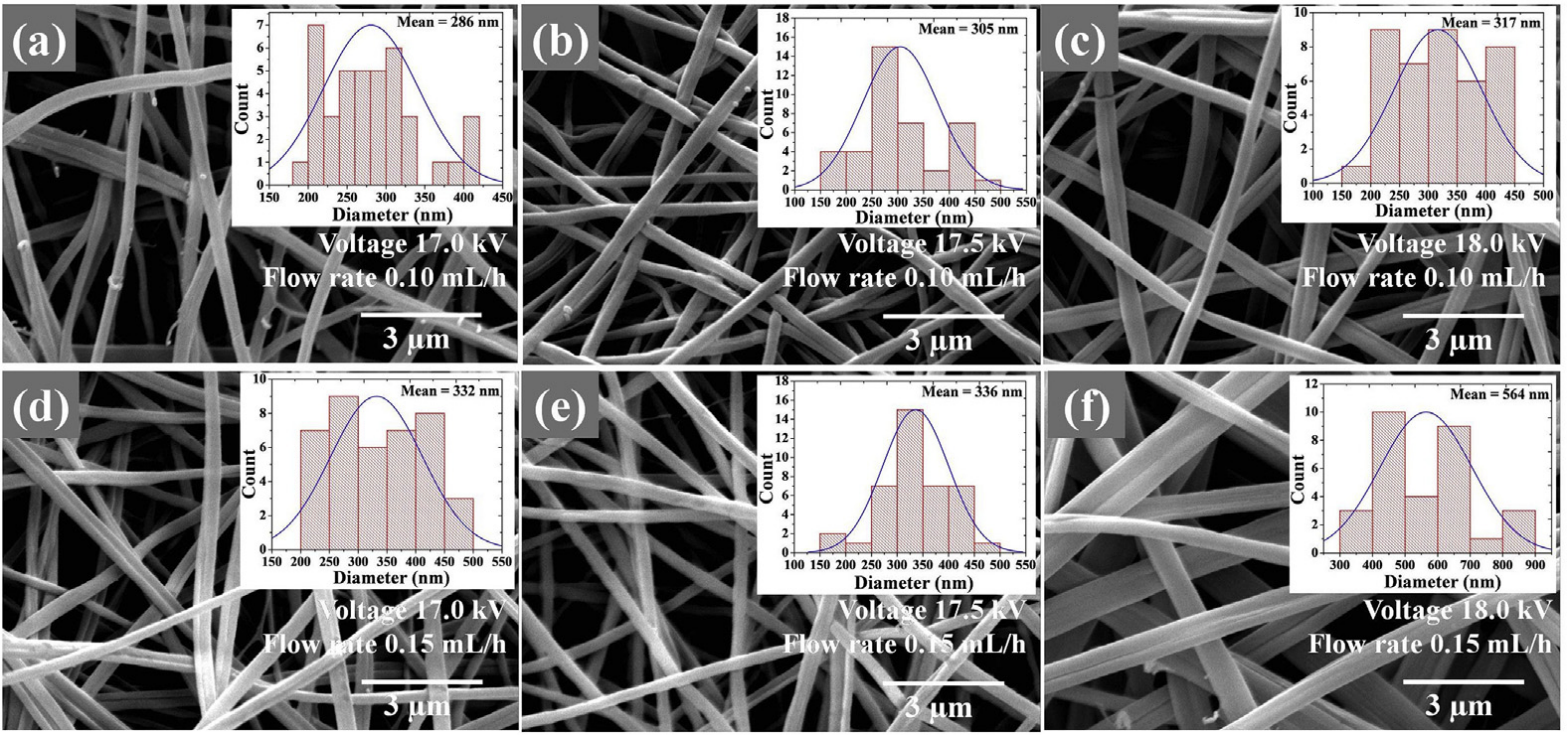
SEM images showing diameters and morphology of the electrospun composites fabricated under various applied voltages and solution flow rates.

**Fig. 2:**
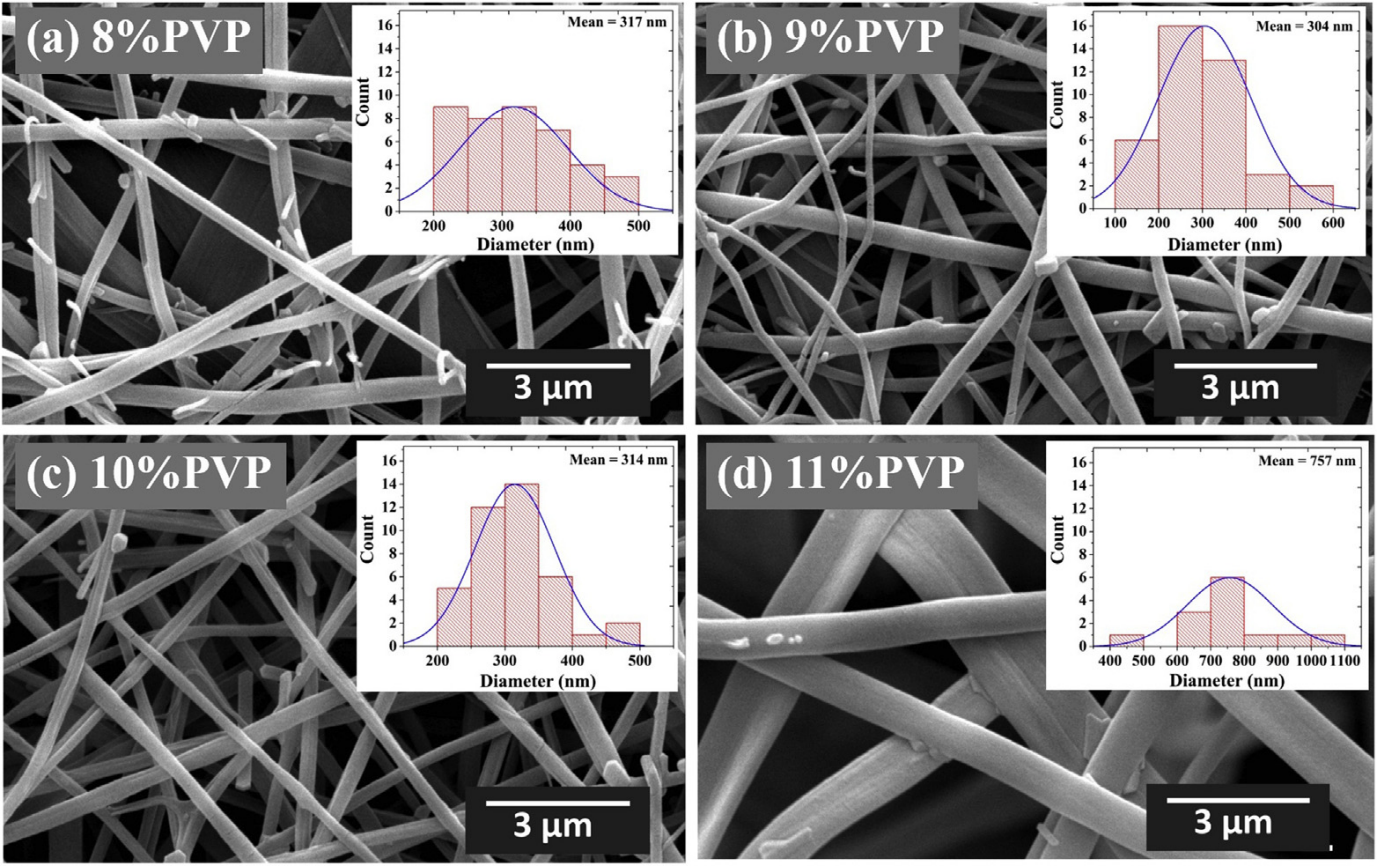
SEM images of electrospun LiFePO_4_ composites from various concentrations of PVP dissolved in (a) 8 wt%, (b) 9 wt%, (c) 10 wt%, and (d) 11 wt% of solution precursor.

**Fig. 3:**
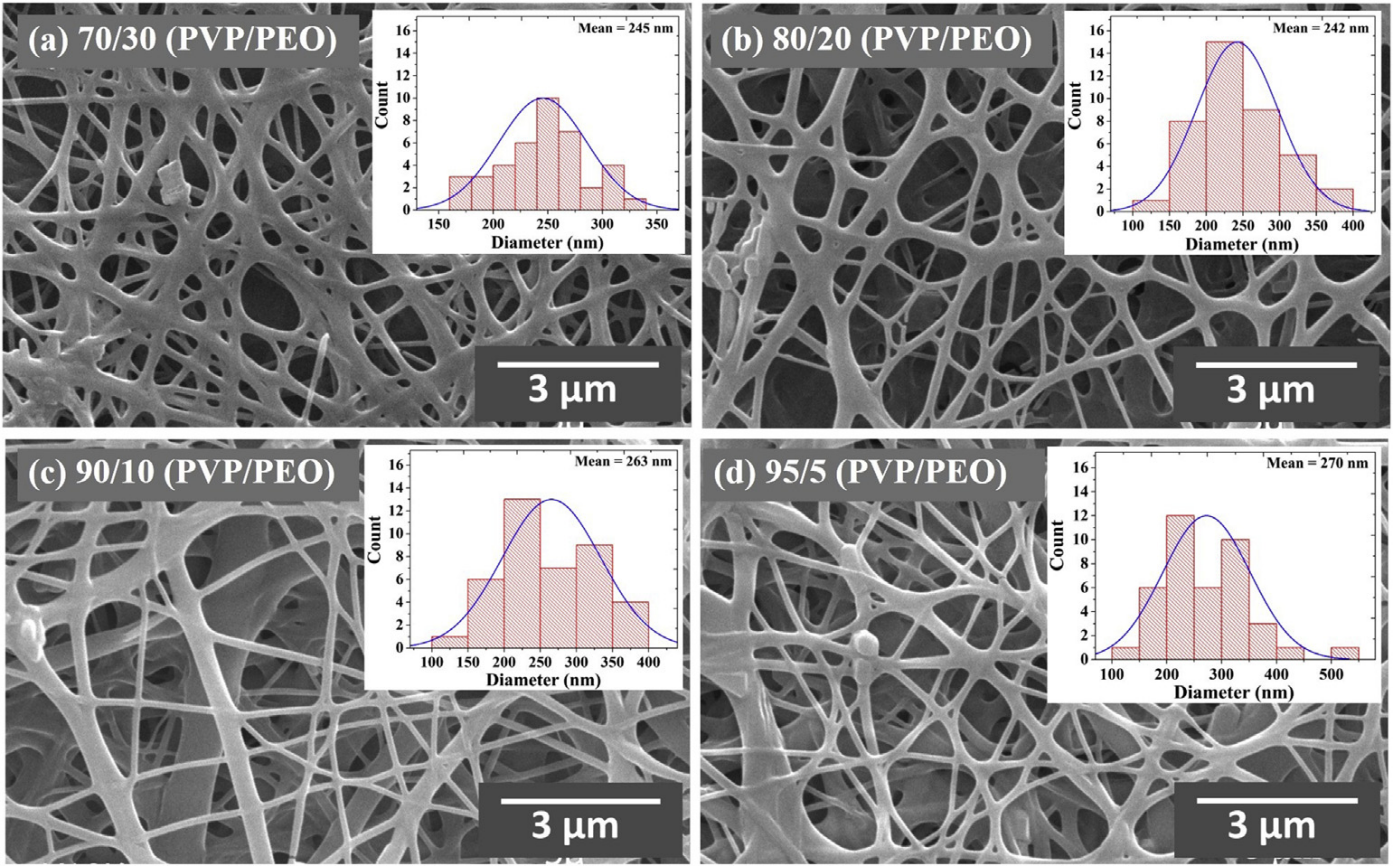
SEM images of electrospun LiFePO_4_ composites from a mixture of PVP/PEO polymers with PVP/PEO weight ratios (w/w%) of (a) 70:30, (b) 80:20, (c) 90:10, and (d) 95:5.

**Fig. 4:**
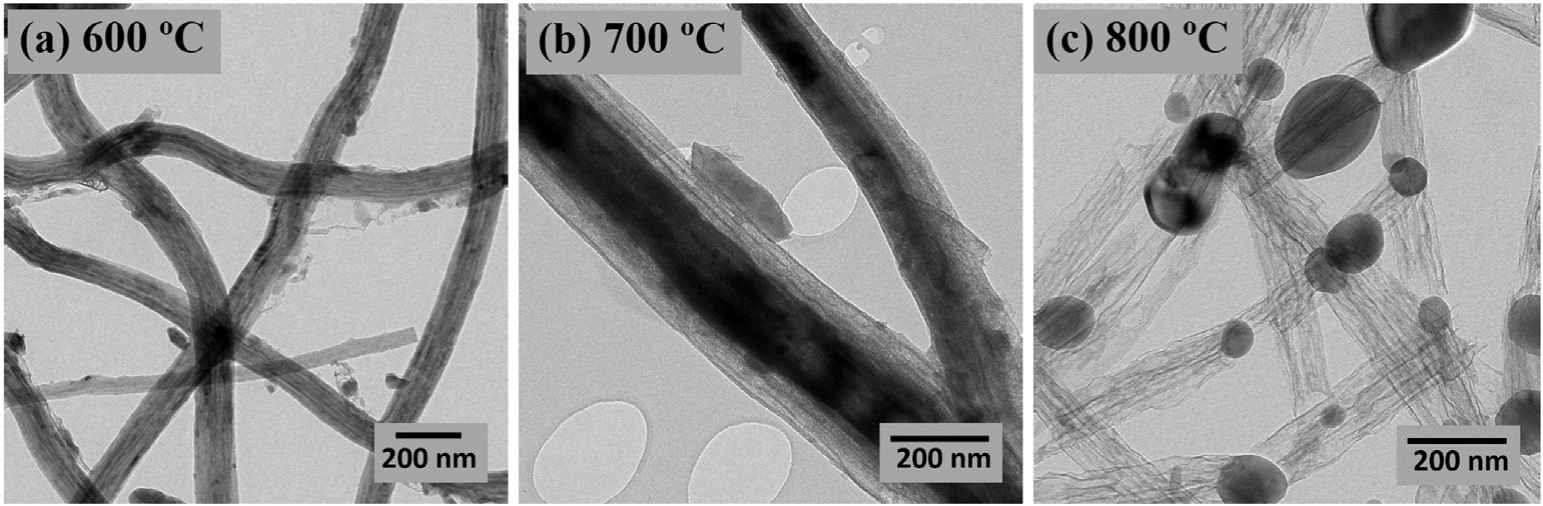
TEM images of electrospun LiFePO_4_ composites obtained at various heat-treatment temperatures of (a) 600 ᵒC, (b) 700 ᵒC, and (c) 800 ᵒC.

**Fig. 5:**
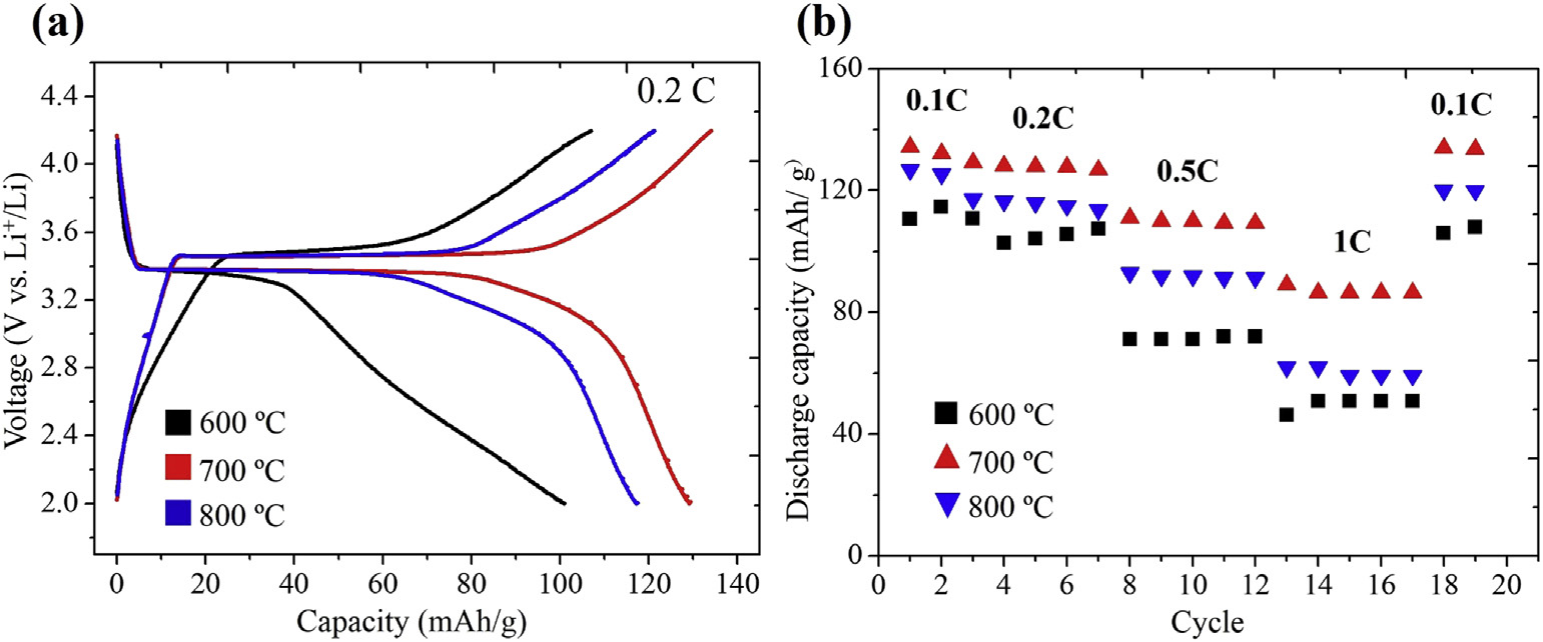
Typical galvanostatic charge-discharge curves (a) and rate capability test at various current densities (b).

The data presented in this study have been derived by fabricating core-shell LiFePO_4_/FeS/C composites fibers using an electrospinning method and subsequent heat-treatment processes. Fig. 1 and Table 1 show the fiber morphologies and diameters obtained with a controllable applied voltage and solution flow rate in an electrospinning process. Fig. 2 and Fig. 3 show the effects of the concentration of PVP (wt%) and mixed PVP/PEO (w/w%) on fiber morphologies and diameters. Fig. 4 shows TEM images of core-shell electrospun LiFePO_4_/FeS/C composites after heat-treatment at various temperatures. Fig. 5a and b shows the voltage profiles and cycle rate properties, respectively, from a half-cell electrochemical test.

**Table 1:** Average diameters of electrospun LiFePO_4_/FeS/C composite fibers.

Flow rate (mL/h)	Voltage (kV)	Average diameter (nm)
0.10	17.0	281
	17.5	305
	18.0	317
0.15	17.0	332
	17.5	336
	18.0	564

## 2 Experimental design, materials, and methods

Electrospinning is a powerful technique that utilizes electrostatic forces to produce continuous fibers and their composites with diameters ranging from 3 nm to over 1 μm from a polymer solution [2], [3]. Many parameters influence the overall process outcome. These include solution parameters (concentration, solubility, solution viscosity, and type of polymer), process variables (applied voltage, the feed rate, the distance between spinneret tip and ground collector), and ambient conditions (temperature and humidity) [4], [5]. Experimental details for the preparation of electrospun LiFePO_4_/FeS/C composites have been previously presented [1]. Briefly, lithium hydroxide monohydrate, iron (II) sulfate heptahydrate, phosphoric acid, and citric acid were added to de-ionized water to prepare a spinning solution. PVP and a mixed PVP/PEO solution were used as the carbon source for fabrication of composite fibers.

### 2.1 The effect of processing parameters on electrospun morphologies and sizes

The effects of processing parameters on the morphology and diameter of electrospun fibers were determined at applied voltages of 17.0, 17.5, and 18.0 kV and solution flow rates of 0.10 and 0.15 mL/h. These observations were made using SEM and are shown in Fig. 1a–f. It can be seen that the smallest fiber diameter is approximately 286 nm at an applied voltage of 17.0 kV and a solution flow rate of 0.10 mL/h. The diameter distribution range obtained from image analysis (Image J software) is 180–416 nm (seen in Fig. 1a). The fiber diameters tend to increase when these parameters are increased, as summarized in Table 1.

### 2.2 The effect of %PVP and the mixed with PVP/PEO (w/w%) on electrospun morphologies and sizes

Generally, the solution properties are one of the most critical factors affecting the characteristics of electrospun fibers. They determine the limiting boundaries for formation of these fibers due to variations in the viscosity and surface tension of the solution [6]. The spinning solutions were prepared by varying the concentration of PVP from 8 wt% to 11 wt%. Fig. 2a–d illustrates the effect of the PVP concentration on the morphology and fiber diameters from SEM. The results show that the lowest concentration of PVP, 8 wt%, had a higher tendency to form beads due to aggregation of solvent molecules. At high concentrations, the solvent molecules are distributed among the entangled chains and their tendency to agglomerate decreases [7]. The average diameter of fibers with 8 wt%, 9 wt%, and 10 wt% of PVP solutions was 317, 304, and 314 nm, respectively. When the concentration reached 11 wt%, the beads disappear while the average fiber diameter increases to around 757 nm. Therefore, in this study, a 10 wt% PVP solution is used to determine an optimum electrospinning condition of the PVP solution. This condition produced a fiber diameter distribution ranging from 225–483 nm and average fiber diameter of 314 nm.

The PEO (M_w_: 100,000 g/mol) was mixed with PVP polymer for use as a precursor solution. The mixed with PVP/PEO (w/w%) solution was directly dissolved in the precursor solution at ratios of 70:30, 80:20, 90:10, and 95:5 (w/w%). Fig. 3 a–d shows SEM images giving the fiber diameters resulting from PEO addition with various PVP/PEO (w/w%) ratios. Increased PEO resulted in a broad range of fiber diameters due to the water-soluble properties of PEO, melting the fibers.

### 2.3 The effect heat-treatment temperature on morphology and electrochemical properties

TEM was used to elucidate the effects of heat-treatment temperature on the characteristic morphology of core-shell electrospun LiFePO_4_/FeS/C composites after calcination at 600 °C, 700 °C, and 800 °C. The observed sample morphologies are shown in Fig. 4a–c. At 600 °C and 700 °C, continuous 1D fiber morphology and a core-shell structure was observed that disappeared completely at temperatures above 700 °C (see Fig. 4b). At higher heat-treatment temperatures, near-equiaxed particle morphologies are observed. These particles are detached from the amorphous carbon shell.

The voltage profiles of the samples are shown in Fig. 5a. The composites fibers with heat-treatments at 600 °C, 700 °C, and 800 °C reveal charge-discharge capacities at a 0.2C rate of 110, 130, and 117 mAh/g, respectively and their coulombic efficiencies are 94%, 97%, and 96%, respectively. The specific capacities in this study are calculated based only on the content of LiFePO_4_ in the material (170 mAh/g). The rate performance of samples at various current densities of 0.1C, 0.2C, 0.5C, and 1C are shown in Fig. 5b. From this figure, it can be seen that the electrospun LiFePO_4_/FeS/C composites with a core-shell structure exhibit excellent cycle rate property.

## Acknowledgement

This work was supported by New Researcher Grants from the National Science and Technology Development Agency (NSTDA), Institute of Nanomaterials Research and Innovation for Energy (IN-RIE) of Khon Kaen University, and the National Nanotechnology Center (NANOTEC), NSTDA, Ministry of Higher Education, Science, Research, and Innovation, Thailand, through its program of Research Network NANOTEC (RNN). R.H. gratefully acknowledges the Science Achievement Scholarship of Thailand (SAST) for financial support.

## Conflict of interest

The authors declare that they have no known competing financial interests or personal relationships that could have appeared to influence the work reported in this paper.
